# Editorial: Regulation of AMPA receptors in brain diseases, from the genetic to the functional level, volume II

**DOI:** 10.3389/fnsyn.2024.1470791

**Published:** 2024-08-28

**Authors:** Laura Jiménez-Sánchez, Tak Pan Wong, Alberto Ouro

**Affiliations:** ^1^Department of Physiology, Universidad de Granada, Ibs.Granada, Granada, Spain; ^2^Basic Neuroscience Division, Douglas Research Centre, Montreal, QC, Canada; ^3^Department of Psychiatry, McGill University, Montreal, QC, Canada; ^4^NeuroAging Group (NEURAL), Clinical Neurosciences Research Laboratories (LINCs), Health Research Institute of Santiago de Compostela (IDIS), Santiago de Compostela, Spain; ^5^Centro de Investigación Biomédica en Red de Enfermedades Neurodegenerativas, Instituto de Salud Carlos III, Madrid, Spain

**Keywords:** hyperexcitation, modeling studies, NMDA receptors, synaptic maturation, synaptic plasticity, social behaviors

In recent years, α-amino-3-hydroxy-5-methyl-4-isoxazolepropionic acid (AMPA) receptors have gained great interest among the scientific community, given their fundamental role in excitatory synapses and their ability to modulate brain function rapidly. Acting as ligand-gated ion channels, AMPA receptor is the main driver of excitatory neurotransmission in the brain. This role is essential for synaptic transmission and plasticity, neuronal activity, and behaviors. Dysfunction or dysregulation of AMPA receptors has been associated with various brain disorders, including neurodegenerative diseases such as Alzheimer's disease and psychiatric disorders such as schizophrenia. This Research Topic further elaborates on the role of AMPA receptors in epilepsy and altered social behaviors, as well as its relationship with N-methyl-D-aspartate (NMDA) receptors during development and synaptic plasticity phenomena.

Neural functioning can be better understood by modeling the physiological mechanisms that underlie it. This context makes mathematical models particularly relevant, but it poses a huge challenge to successfully and coherently integrate what is known about the molecular, synaptic, and neuronal components involved and to transfer all this information into large simulations. In this Research Topic, Dainauskas et al. presented a model to understand synaptic plasticity at hippocampal CA3-CA1 synapses. Given the plasticity of AMPA receptors is largely mediated by NMDA receptors, the authors emphasized the roles of GluN2A and GluN2B NMDA receptor subunits in their model. Interestingly, their model is able to predict synaptic changes based on voltage-dependent mechanisms. Validated against experimental data, it showed how the GluN2B subunit influences learning rules and synaptic strength, providing insights into both healthy brain function and pathological conditions.

Understanding rules that govern the formation of synaptic circuitry could shed light on the pathogenesis of neurodevelopmental disorders. Chen et al. highlighted the importance of NMDA receptors in synaptic maturation and function. Although AMPA receptors and NMDA receptors showed smooth distributions across dendrites, short periods of Ca^2+^ influx resulted in a rapid clustering of NMDA receptors, followed by an accumulation of Ca^2+^/calmodulin-dependent protein kinases (CaMKII) and AMPA receptors. These results suggest that glutamate-triggered signaling contributes to the maturation of young synapses through sequential recruitment of NMDA and AMPA receptors.

Hyperexcitation of a small number of glutamatergic neurons is responsible for inducing epileptic seizures. Zinchenko et al. demonstrated a novel mechanism of calcium-permeable AMPA receptors in GABAergic neurons for triggering seizures: by releasing GABA onto other GABAergic neurons, GABA activated GABA_B_ receptors and potassium channel Kv7, which in turn reduced the activity of postsynaptic GABAergic neurons. Reduced activity of these GABAergic neurons would facilitate seizures by the disinhibition of glutamatergic neurons. These results identify new targets for the treatment of epilepsy and other neurodegenerative diseases that are related to the hyperactivation of glutamatergic neurons.

Finally, Xu et al. reviewed the state of knowledge about the role of AMPA receptors in social behaviors. They highlighted the role of different AMPA receptor subunits in social behaviors like aggression or sociability. In addition, they summarized the role of AMPA receptors in abnormal social behaviors in brain diseases such as schizophrenia and autism spectrum disorders. A better understanding of the contribution of AMPA receptors to social behavior would allow the identification of new therapeutic targets for treating these pathologies.

Together, these papers illustrated a wide range of mechanisms that underlie the maturation, plasticity and network properties of AMPA receptors. The abnormality of these mechanisms could underlie synaptic pathologies in neurodevelopmental disorders and epilepsy. In addition, an increased understanding of the role of AMPA receptors in behaviors creates opportunities for targeting these receptors in treating behavioral deficits in brain diseases. Further examination of the contribution of AMPA receptors to brain diseases remains a promising avenue for discovering novel treatments.

